# Supplementary material for: Parental experience of the diagnostic process and its role in the decision to terminate pregnancy due to fetal abnormality; A qualitative interview study
Source: PLoS One. 2025 Jun 6;20(6):e0325690. doi: 10.1371/journal.pone.0325690 (PMC12143557; doi:10.1371/journal.pone.0325690)
Supplement: Attachment — Submitted filename: Response Letter_R1.docx [file pone.0325690.s002.docx]

## Response to decision letter

Dear Mr. Okonta, Academic Editor, PLOS One

First, we wish to thank the reviewers for positive and constructive comments on our manuscript. We have revised the paper in accordance with the reviewers' suggestions and believe it has improved the manuscript.

Below we address the comments and explain how we have aimed to accommodate them in the revision of the manuscript. All reference to line numbers are in the track changes version of the revised manuscript.

We hope that the revision meets your expectations and look forward to hearing from you.

On behalf of the authors,

Best regards,

Stina Lou

| Journal Requirements |  |
| --- | --- |
| 1. Please ensure that your manuscript meets PLOS ONE's style requirements, including those for file naming | We have revised the manuscript in accordance with PLOS ONE's formatting requirements, including title page, table formatting, file naming and manuscript headings |
| 2.  We note that the grant information you provided in the ‘Funding Information’ and ‘Financial Disclosure’ sections do not match.  When you resubmit, please ensure that you provide the correct grant numbers for the awards you received for your study in the ‘Funding Information’ section. | We apologize for this inconsistency. |
| 3. Thank you for stating the following financial disclosure:   [This work was funded by University Hospital of Southern Denmark (grant#23) and by Aarhus University Hospital, (grant#SVP101121 and SVP040723).].  Please state what role the funders took in the study.  If the funders had no role, please state: ""The funders had no role in study design, data collection and analysis, decision to publish, or preparation of the manuscript.""  If this statement is not correct you must amend it as needed.  Please include this amended Role of Funder statement in your cover letter; we will change the online submission form on your behalf. | Updated funding statement:  This work was funded by University Hospital of Southern Denmark (grant#23) and by Aarhus University Hospital, (grant#SVP101121 and SVP040723).The funders had no role in study design, data collection and analysis, decision to publish, or preparation of the manuscript.  Thank you very much for making this change in the online submission form on our behalf. |
| 4. We note that you have indicated that there are restrictions to data sharing for this study. For studies involving human research participant data or other sensitive data, we encourage authors to share de-identified or anonymized data. However, when data cannot be publicly shared for ethical reasons, we allow authors to make their data sets available upon request.  Before we proceed with your manuscript, please address the following prompts:  a) If there are ethical or legal restrictions on sharing a de-identified data set, please explain them in detail (e.g., data contain potentially identifying or sensitive patient information, data are owned by a third-party organization, etc.) and who has imposed them (e.g., a Research Ethics Committee or Institutional Review Board, etc.). Please also provide contact information for a data access committee, ethics committee, or other institutional body to which data requests may be sent.  Please update your Data Availability statement in the submission form accordingly. | We have updated and provided more information to the Data Availability Statement:  The data underlying this study consist of in-depth qualitative interviews containing potentially identifiable and sensitive personal information. Due to the highly individual nature of the material, full transcripts cannot be fully anonymized, and public sharing would not comply with the ethical approval granted for this study. Participants consented only to external data sharing in anonymized form.  Data access requests may be directed to DEFACTUM, Central Denmark Region, at defactum@rm.dk, and will be considered for researchers who meet the criteria for access to confidential data in accordance with the Central Denmark Region’s data protection policies. |
| 5. In the online submission form, you indicated that [For this study, participants only consented to external data sharing in anonymized form. Since full transcripts cannot be fully anonymized due to the highly individual context, the transcripts can only be made available upon reasonable request and special conditions may apply. Any requests concerning data access can be directed to [defactum@rm.dk](mailto:defactum@rm.dk)].  All PLOS journals now require all data underlying the findings described in their manuscript to be freely available to other researchers, either 1. In a public repository, 2. Within the manuscript itself, or 3. Uploaded as supplementary information.  This policy applies to all data except where public deposition would breach compliance with the protocol approved by your research ethics board. If your data cannot be made publicly available for ethical or legal reasons (e.g., public availability would compromise patient privacy), please explain your reasons on resubmission and your exemption request will be escalated for approval. | Thank you for your comment. As detailed in the updated Data Availability Statement, the data for this study consist of in-depth qualitative interviews that cannot be fully de-identified due to the highly personal and contextual nature of the material. Participants consented only to anonymized data sharing, and full public deposition would breach this consent.  To balance transparency with ethical obligations, data will be made available upon reasonable request and subject to conditions that ensure participant confidentiality. Requests can be directed to DEFACTUM, Central Denmark Region, at defactum@rm.dk. We hope that PLOS ONE will accept this exemption in accordance with its policy on sensitive data and ethical restrictions. |
| 6.  Please include a caption for figure 1. | We have uploaded a TIFF file of the figure with caption |
|  |  |
| Reviewer #1 |  |
| 1) hermeneutical and phenomenological approach needs to be explained more. | This is a very valid point. We have elaborated on our approach, L81-86 |
| 2) As authors have used semi-structured interviews, it should be explained that how interview guide was developed? | Good point, and we have added more information on this, L123-5 + L128-32 |
| 3) Justify that how much time after abortion the interviews have been done? | Sure! The timing of the interviews were based on participant preference and convenience, which we have added L126-8 |
| 4) Implications of study should be extended more | We have added a ‘Clinical Implications’ section to the Discussion, L450-8 |
| 5) Adding some more demographic and clinical characteristics of participants is worthy. | Thank you for this suggestion, which we have discussed in the author group. However, given the rarity of the diagnoses and therefore characteristics of the participants, providing more detail would compromise their anonymity. We hope for your understanding. |
|  |  |
| Reviewer #2 |  |
| Reviewer #2: The manuscript is well written and recommended for it's technicality and it's originality. But I would suggest the author make a tabular thematic analysis between the two themes as stated by the author. | Thank you for the positive evaluation of our manuscript! Also, providing a graphical display of the two themes is a very useful suggestion. We have added Table 2 to the manuscript, see manuscript page 10. |
|  |  |
|  |  |
|  |  |
